# Emergency trauma theatre utilisation – the St George’s Hospital, London experience

**DOI:** 10.1186/1757-7241-22-S1-P4

**Published:** 2014-07-07

**Authors:** Alexander J Hills, Alexander Clarke, Christoph EA Hartmann, Caroline B Hing

**Affiliations:** 1 St George’s University, London, UK; 2 Charing Cross Hospital, London, UK

## Background

The Royal College of Surgeons of England encouraged the creation of singly dedicated 24-hour emergency theatres in 1997; emergency operating was previously largely determined by theatre availability rather than clinical need. Shortly after this St George’s Hospital, London designated one of its three trauma theatres as an ‘Acute Emergency Theatre’ (AET) and implemented a policy to maintain access to the AET within one hour’s notification for all acute trauma emergencies.

On review it was felt that the system underutilised scarce theatre space and limited the repertoire of surgeries that could be performed. A more efficient use of theatre space was therefore sought. The AET was changed from a fixed physical entity to a nominal entity; where by the AET could be any of the three major trauma theatres, as long as one of them was available within one hour.

## Methods

Theatre usage across all three major trauma theatres was prospectively audited three months before and three months after the implementation of the new nominal system following a one-month familiarisation period. An anonymous, Likert based questionnaire was completed by ten of the eleven overseeing trauma consultants to review concerns and ensure our standard of care was maintained.

## Results

1325 consecutive operations were performed, 660 operations before and 665 after the systems implementation (p=0.45). A 32% increase in operating hours (216 to 284 hours, p=0.01) within the original AET (Theatre 1) was achieved, and a 10% increase in operating hours (1102 to 1223 hours, p=0.19) seen across all three theatres. There were no serious incident reports regarding access to theatres following implementation of the new system or any compromise to patient care.

## Conclusions

Implementing a nominal emergency theatre, rather than utilizing a dedicated emergency theatre allows improved theatre utilization, without adverse event.

